# Optimizing daylight in west-facing facades for LEED V4.1 compliance using metaheuristic approach

**DOI:** 10.1038/s41598-023-49025-0

**Published:** 2023-12-11

**Authors:** Pham Vu Hong Son, Vo Thi Bich Huyen

**Affiliations:** Faculty of Civil Engineering, Ho Chi Minh City University of Technology (HCMUT), Vietnam National University (VNU-HCM), Ho Chi Minh City, Vietnam

**Keywords:** Civil engineering, Computational science

## Abstract

This study introduces an optimized design approach for west-facing room façades to improve daylighting while adhering to LEED v4.1 sustainability criteria. Employing parametric modeling, metaheuristic optimization, and validated daylight simulations, the research highlights the African Vulture Optimization Algorithm's success in achieving 100% LEED compliance and superior performance over random models in daylight sufficiency and glare reduction. Light-colored materials and transparent glazing emerged as beneficial for LEED points. Despite computational limitations and the need for empirical validation, this method offers architects versatile and sustainable design solutions. Comparative analysis reveals the algorithm's strong performance, although opportunities exist for refinement. Future research directions include contrasting this algorithm with other optimization methods, focusing on empirical backing, assessing environmental and human-centric impacts, adapting to varied building types and conditions, and examining diverse geographical and material factors. This work advances daylight-integrated façade design, suggesting a more comprehensive framework for building performance optimization.

## Introduction

In recent times, green building design has become increasingly prominent in architectural practices worldwide^1^. Rating systems such as Leadership in Energy and Environmental Design (LEED) offer benchmarks for sustainable building standards^2^. A critical aspect of this sustainability focus is the optimization of daylighting, which can both increase visual comfort and decrease electricity consumption^3^. However, reconciling the advantages of daylight with potential issues such as glare and overheating presents complexities, especially for west-facing facades exposed to western sunlight. Building facades play a significant role in shaping a structure's energy and daylighting characteristics, influencing energy consumption through the modulation of heat, light, and airflow^4^. Effective daylighting can decrease the reliance on artificial lighting, promote even light distribution, and enrich views, which can contribute to occupant well-being. Metrics like sDA (Spatial Daylight Autonomy) and ASE (Annual Sunlight Exposure) serve as standard tools in the industry to measure daylight performance^5^. The ongoing objective is to optimize daylight to enhance energy efficiency and comfort while adeptly controlling glare to ensure visual comfort. While numerous studies have addressed facade optimization in various contexts, there remains a specific gap in developing an optimization approach tailored for west-facing facades that aligns with the stringent requirements of the LEED v4.1 daylight criteria.

**Figure 1:**
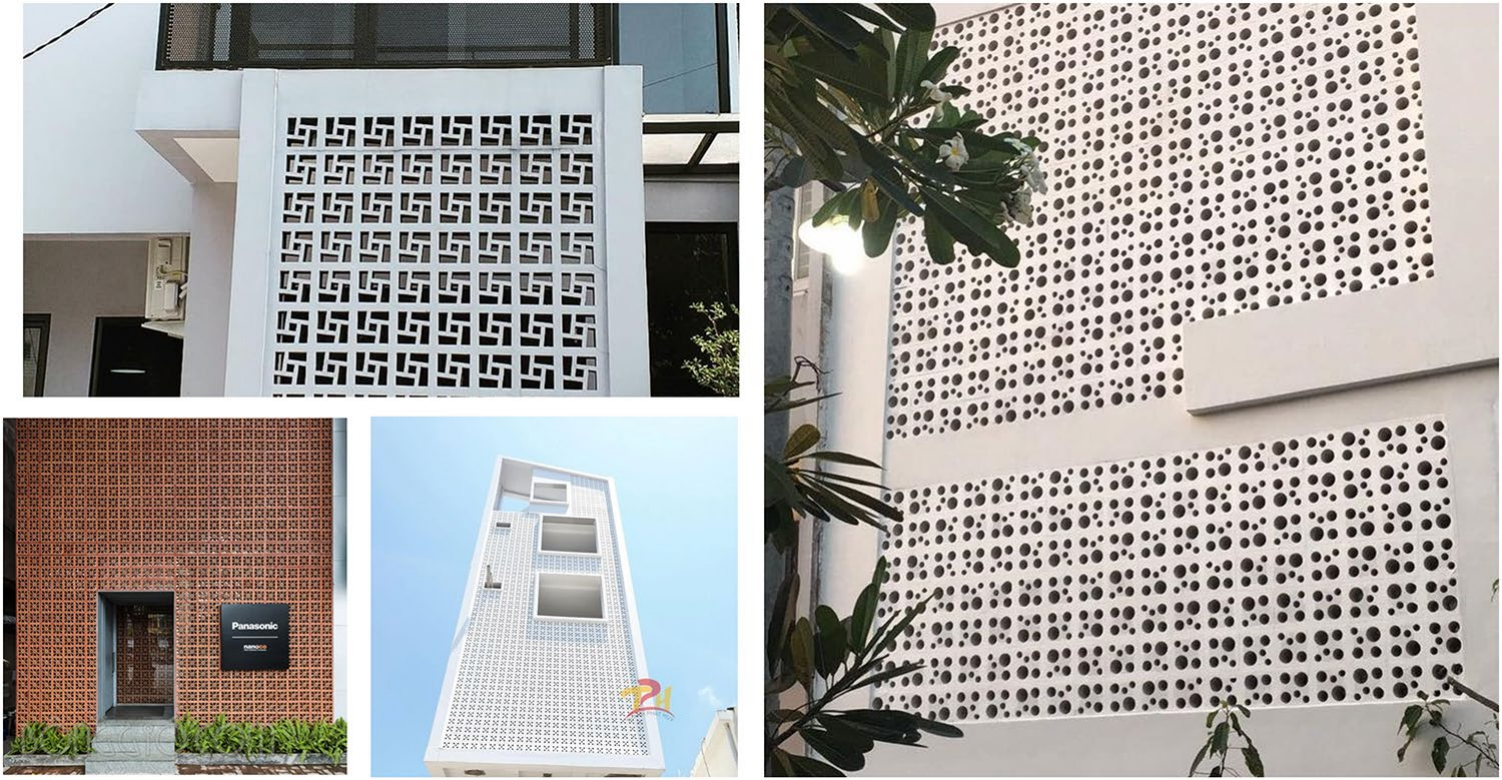
Examples of buildings that utilize ventilation bricks.

In Asia, pattern ventilation tiles, a culturally significant architectural feature, have re-emerged as a popular design trend, as illustrated by some examples in Fig. 1. This paper aims to contribute to this tradition by proposing an optimization approach for west-facing facades to enhance daylight quality while aligning with LEED v4.1 daylight criteria^6^. The objective of this work is not to identify an optimal solution, but rather to investigate an approach that can generate varied and advantageous design configurations, improving visual appeal while sustaining compliance with LEED criteria. Furthermore, the proposed methodology is intended for practical application, consisting of a modular and parametric model utilizing commercially available materials. The techniques harness existing algorithms and software platforms to reconcile the advantages of daylighting with the complications it poses.

## Literature review

### Daylighting metrics

Prior to reviewing previous relevant research, it is crucial to elucidate the daylighting metrics that will guide this investigation.

The Leadership in Energy and Environmental Design (LEED) green building certification system was selected for this study owing to its broad recognition as an industry benchmark^2^. More specifically, version 4.1 of LEED was employed, which emphasizes passive design and daylighting approaches. The integration of natural daylight provides several benefits, including:

- Reduced energy consumption through lessened reliance on artificial lighting^3^. By harnessing natural light sources, buildings can decrease electricity requirements.
- Enhanced occupant experience through exposure to natural illumination^7^. Access to daylight has been correlated with improved wellbeing and satisfaction among building inhabitants.
- Heightened alertness and productivity^7^. Natural light exposure assists in regulating circadian rhythms and cognitive performance.
- Potentially lowered absenteeism resulting from these factors. By generating more inviting and engaging spaces with daylight, employee attendance and retention may also increase.

The metrics of sDA and ASE were selected for this study as they align with LEED v4.1 daylighting criteria and have become industry standards for assessing daylight performance. sDA indicates the percentage of a space that has sufficient daylight levels for occupants, while ASE helps identify potential glare issues from excessive direct sunlight^5^. Although other metrics like daylight factor, daylight autonomy, and useful daylight illuminance were considered initially, sDA and ASE were ultimately chosen due to their stronger alignment with LEED requirements.

In particular, LEED certified projects must achieve benchmarks:

- **Spatial Daylight Autonomy (sDA)** The percent of floor area that meets a minimum daylight illuminance level (300 lx) for at least 50% of occupied hours annually. To earn LEED points, projects must achieve sDA300/50% for at least 40% of the occupied floor area (1 point), 55% (2 points), or 75% (3 points). This study targeted the highest level of 75% sDA^6^.
- **Annual Sunlight Exposure (ASE)** The percent of floor area that receives at least 1000 lx for at least 250 occupied hours per year. LEED v4.1 stipulates an ASE1000/250 upper limit of no more than 10% of the occupied space^6^.

### Previous studies

Numerous studies have delved into the topic of facade optimization. ElBatran et al.^8^ investigated the design of an optimal double skin south-facing façade for Egypt's hot climate to align with LEED requirements. However, their research did not encompass the influence of façade geometry, reflectivity, and color on daylight performance. Similarly, Zhang and Ji^9^ employed parametric tools, such as Rhino and Grasshopper, to optimize apartment window designs using genetic algorithms, yet LEED sustainability metrics were not a focal point of their study.

Glassman and Reinhart^10^ explored the impact of future climate scenarios on facade optimization using parametric design and simulation tools. Their study, while insightful, focused on energy and carbon emissions as the main criteria, neglecting the human-centric aspects of facade performance. Daylight quality, visual comfort, and occupant satisfaction were not considered in their analysis.

Shan and Junghans^11^ developed an evolutionary algorithm called adaptive radiation to optimize building facade design for different climates. Their approach modifies a simple genetic algorithm to achieve near globally optimal solutions with substantially reduced computational cost. Although their methodology efficiently optimizes facades for energy performance related to cooling, heating, and artificial lighting requirements, it does not account for human-centric factors that impact facade design.

Bakmohammadi and Noorzai^12^ proposed a framework to assist architects in evaluating classroom designs, with an emphasis on thermal comfort and sustainability. In a different approach, Hosseini et al.^13^ highlighted the role of facades in enhancing daylight performance in architectural designs. Their model, while intricate, was tailored for a specific building type, potentially limiting its broader applicability. LEED metrics were not a component of their analysis.

Wang et al.^14^ focused on refining a composite shading system for classrooms in Nanchang, China, using 3D parametric modeling and the NSGA-II algorithm. However, their study did not incorporate sustainability metrics. Tabadkani et al.^15^ assessed visual comfort by contrasting dynamic facade systems with static ones, targeting the improvement of daylight performance. In another study, Fang and Cho^16^ delved into facade optimization across different climates, analyzing various design variables for a small office building in cities like Miami, Atlanta, and Chicago.

Do and Chan^17^ offer valuable insights into daylight and glare control for multi-sectional facades. Our research aims to complement their work by directly addressing LEED v4.1 daylight criteria, with a focus on visual comfort and daylight sufficiency. While Do and Chan's study provides a strong foundation, the alignment with LEED standards in our work allows for more direct applicability to LEED-targeted projects. Additionally, by optimizing west-facing facades with ventilation bricks, an architectural feature common in Asia, our study explores a practical application while adhering to sustainability principles. This study may contribute meaningfully to the field of sustainable building design through its blended approach, aligning established LEED metrics with regionally relevant designs.

Kizilörenli and Tokuç^18^ developed a novel responsive façade system targeting daylight optimization for west-facing façades. Their parametrically modeled system focused on maximizing Spatial Daylight Autonomy (sDA) while keeping Annual Sunlight Exposure (ASE) under 10% to reduce visual disturbance. Employing tools like Rhinoceros 3D, Grasshopper, Climate Studio, and Octopus, they effectively improved daylight data from initial models, achieving better user comfort levels. Their approach, although parallel to ours in optimizing daylight control, diverges in several aspects. Notably, their method did not incorporate LEED standards, lacked material variation, and compared outcomes only to a base scenario with no façade, rather than exploring a range of real-life façade designs. This approach, while yielding a range of solutions, omits a direct comparison with commonly used façade designs, potentially limiting the broader applicability of their findings in real-world scenarios.

Prior studies have provided important insights into facade optimization, yet some areas remain less explored. Many studies did not examine the integration of facade geometry, reflectivity, and color for daylight performance and occupant satisfaction. Others emphasized energy use and thermal comfort without incorporating LEED metrics. Some works focused on specific buildings or contexts. Building on these contributions, the current study aims to optimize west-facing facades for LEED v4.1 daylight criteria. We evaluate visual comfort, daylight sufficiency, and ventilation bricks—an architectural feature common in Asia. By aligning with LEED standards and focusing on regionally relevant elements, we hope to connect theory and practice, adding to knowledge on sustainable facade design.

### Nature-inspired metaheuristics: exploring AVOA's potential and performance

Metaheuristics have garnered significant attention in the realm of computational intelligence for their ability to offer solutions to complex optimization problems that traditional algorithms struggle with^19^. These algorithms, often inspired by natural or sociological phenomena, aim to strike a balance between exploration (searching new areas in the solution space) and exploitation (refining current solutions). The ingenuity of these techniques lies in their emulation of specific behaviors observed in nature or society, effectively leveraging the inherent wisdom of these systems.

In line with this growing interest in bio-inspired optimization techniques, researchers have recently begun applying such algorithms to real-world optimization problems across various domains. For instance, Pham, Nguyen Dang, et al.^20^ introduced an enhanced SCA that integrates roulette wheel selection with OBL, demonstrating superior performance over traditional optimization algorithms in various engineering optimization contexts. Son and Nguyen Dang^21^ presented the MVO model as an effective tool for addressing time–cost optimization issues in construction project management, surpassing other techniques in small-scale applications. Pham et al.^22^ articulated a proficient scheduling optimization technique for ready-mix concrete (RMC) truck dispatches. At its core is a novel hybrid swarm intelligence algorithm fusing the grey wolf optimizer (GWO) with the dragonfly algorithm (DA). The resultant algorithm excels in performance compared to its standalone counterparts and heralds a leap in multi-independent batch plant cooperation for refined RMC deliveries in construction sectors. Son and Nguyen Dang^23^ showcased a composite model named the hybrid multi-verse optimizer (hDMVO), which integrates MVO and the sine cosine algorithm (SCA). This model is adept at managing discrete time–cost trade-off (TCTO) quandaries in construction project orchestration. Its prowess shines through benchmark evaluations and its knack for devising superior solutions in large-scale TCTO scenarios for intricate projects.

For repeating tasks with several concurrent instances, the adaptive selection slime mould algorithm (ASSMA) is presented by Son and Khoi^24^. Son and Khoi also provides the mutation-crossover slime mold algorithm (MCSMA) for balancing time, cost, quality, and work continuity in a specific building project^25^. In addition, there has been extensive research in the field of artificial intelligence. Son and Nam ^26^ employed Hybrid Sine Cosine Optimization to solve the Transport Project. Hybrid multi-verse optimizer model for a sizable discrete time–cost trade-off problem, by PVH Son and NDN Trinh^23^. To address the drawbacks of the GWO algorithm, Son and Trang^22^ introduces HDGM, an unique hybrid optimization model combining the dragonfly algorithm and the grey wolf optimizer. P Vu-Hong-Son et al.^27^ proposed project schedule optimization according to limited resources using dependency structure matrix and whale optimization algorithm. Son and Lien^28^ seeks to advance a Blockchain-based approach for meeting small- and medium-sized project delay resolution demands in a prompt and transparent manner.

In addition to the array of bio-inspired algorithms already discussed, researchers have also begun examining the foraging behaviors of vultures as inspiration for optimization techniques. The African Vulture Optimization Algorithm (AVOA)^29^, draws inspiration from vultures' adaptive foraging behaviors. AVOA replicates vultures' success in foraging by considering hunger levels, physical capabilities, flight adaptability, and inter-vulture dynamics. By synthesizing these factors, AVOA offers a robust optimization technique for complex problem landscapes, mirroring vultures' adeptness in searching for nourishment under varying conditions. Key features of AVOA encompass modeling hunger as solution quality, physical strength as exploration radius, adaptive flight through adjustable parameters, and conflict avoidance via separation distance. We have considered the AVOA as our main candidate algorithm for this study, aiming to maximize visual comfort metrics, for the following reasons:

- Recent development: AVOA, introduced in 2021^29^, is a newly developed algorithm undergoing active refinement^30–35^, offering opportunities for further improvement and application expansion.
- Documented efficiency: Preliminary studies have demonstrated AVOA's consistently impressive performance, often surpassing established metaheuristic algorithms on benchmark optimization tasks^29,35–37^.
- Versatility: The algorithm exhibits versatility in addressing a wide range of optimization challenges, including those with multiple constraints and complex nonlinear problems^35–37^.

In exploring the potential and performance of the AVOA for optimizing façade designs to enhance daylight performance, it is acknowledged that the reasons for choosing AVOA, as outlined earlier, carry a degree of subjectivity. To assess the validity of these reasons and to gain a broader perspective on AVOA's performance, this study will also conduct a preliminary comparison with two well-established metaheuristic algorithms: the Genetic Algorithm (GA) and the Grey Wolf Optimizer (GWO).

This comparative analysis is not intended to be an exhaustive or definitive evaluation of these algorithms. Instead, it aims to provide an initial insight into their relative efficiency in the context of facade optimization for daylight sufficiency and LEED compliance, particularly under the constraints of our limited hardware resources. By acknowledging these limitations, this comparison seeks to offer an insight into the performance of these algorithms in our specific setting, rather than delivering a comprehensive and fully validated assessment. Thus, the primary objective is to explore whether AVOA's selection based on the earlier detailed criteria holds up when compared with GA and GWO in a constrained computational environment.

The Genetic Algorithm (GA), a popular search heuristic that simulates the process of natural selection, was first proposed by Holland^38^. However, the groundwork for GA can be traced back to 1975, marking its deeper roots in optimization theory^39^. This algorithm is widely used for solving complex optimization problems and evolves solutions over generations^40^. Its adaptability and robustness make GA an appropriate benchmark for comparison, demonstrating its long-standing relevance and efficacy in various optimization contexts.

The Grey Wolf Optimizer (GWO), inspired by the social hierarchy and hunting strategy of grey wolves, is a powerful metaheuristic algorithm introduced more recently in 2014^41^. Its ability to explore and exploit the solution space effectively has made it a popular choice in various optimization contexts. This popularity can be attributed to its simplicity, ease of use, flexibility, scalability, and its special ability to balance exploration and exploitation during the search process^42^. As a more modern benchmark, GWO serves alongside the classic GA for comparison with the AVOA, providing a contemporary perspective in evaluating the efficacy of new optimization methods.

In addition to offering preliminary insights into the performance of the AVOA, the GA, and the GWO in optimizing daylight sufficiency in façade designs, this study also seeks to explore the nature of design solutions proposed by each algorithm. A critical aspect of this exploration is understanding the diversity of the solution space each algorithm navigates and the specific preferences or tendencies they exhibit in selecting materials and designs.

## Methodology

To develop an effective optimization approach for west-facing facades, our methodology integrates parametric modeling, a metaheuristic optimization algorithm, and high-fidelity daylight simulations. This allows the exploration of diverse design configurations to identify solutions that enhance daylight performance aligned with LEED v4.1 sustainability standards. The parametric model enables flexibility through key variables like facade geometry, materials, and glazing properties. We employ the African Vulture Optimization metaheuristic algorithm for its strong empirical performance in complex optimization tasks. Validated ClimateStudio simulations provide accurate analysis of daylight metrics for proposed configurations. By strategically combining these capabilities, the methodology aims to generate optimized west-facing facade designs that maximize indoor daylight quality and visual comfort. The goal is an adaptable optimization technique that offers architects expanded design possibilities while ensuring adherence to green building best practices. The following sections delve deeper into the algorithm, software tools, simulation parameters, and metrics underpinning this approach.

### African Vultures Optimization Algorithm (AVOA)

**Figure 2:**
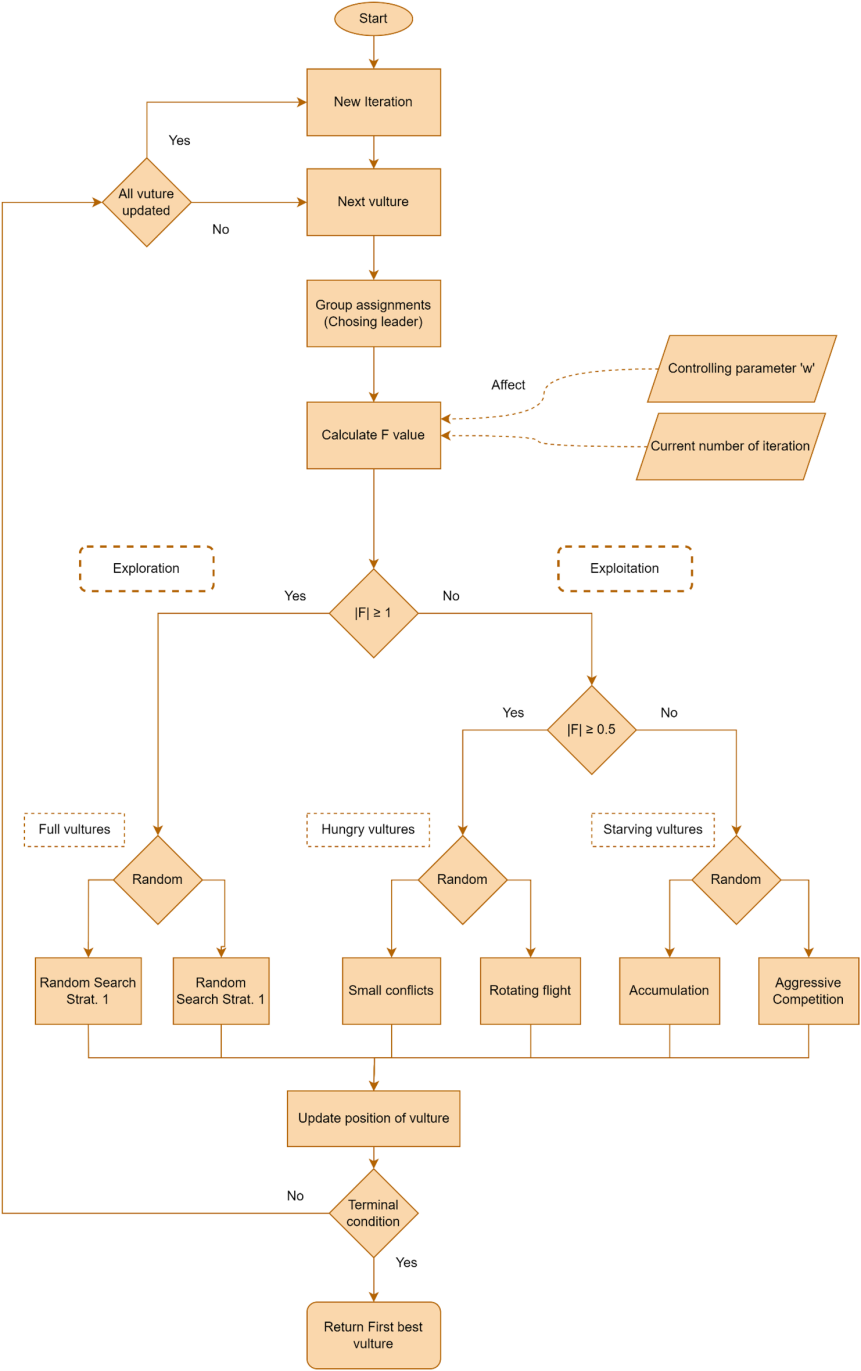
Overview of African Vulture Optimization Algorithm.

In the following section, we provide a concise overview of AVOA as originally developed and presented by Abdollahzadeh et al.^29^, including the key formulas and equations from their research. This summary aims to succinctly elucidate the algorithm's fundamental components and operational mechanisms for the reader. Figure 2 illustrates AVOA's process flow in a visual flowchart, spanning from initialization to termination criteria.

#### Initialization

Population initialization: Begin by determining the number of vultures (N) relevant to the problem at hand. Create an initial population consisting of N individuals.

#### Step one: Determination of optimal vulture selection within a group

After initializing the population, the fitness of all solutions is calculated. The solution with the highest fitness is the optimal vulture in the first group, and the second highest fitness solution is the optimal vulture in the second group. The remaining solutions are directed towards the best solutions in each group using Eq. (1). This process repeats each iteration by recomputing the entire population's fitness.

1 $$\mathrm{R}\left(\mathrm{i}\right)=\left\{\begin{array}{c}{BestVulture}_{1}if {P}_{i} =L1\\ {BestVulture}_{2}if {P}_{i} =L2\end{array}\right.$$

Equation (1) determines the probability of selecting the designated vultures to direct other vultures towards one of the best solutions in each group. Parameters L1 and L2, measured before the search, have values between 0 and 1 that sum to 1. Equation (2) establishes the selection probability of the best solution in each group using the Roulette wheel method.

2 $${P}_{i}=\frac{{\mathrm{F}}_{i}}{\sum_{i=1}^{n}{F}_{i}}$$

#### Step two: The rate of vulture starvation

Satiated vultures fly farther; hungry ones struggle and get aggressive. Equation (4) models this behavior, signaling when to shift from exploration to exploitation based on satiety rates. In these equations, *F* is the vulture's satiation, *iteration*_i_ is the current cycle, and *maxiterations* is the total cycles. *z* and *h* are random numbers affecting the state of the vulture.

Optimization problems do not guarantee precise global solutions, risking convergence to local optima. Equation (3) mitigates this by enhancing adaptability. Initial iterations of the AVOA focus on exploration, while later ones execute exploitation. The goal is to tweak Eq. (3) to oscillate between these phases, enhancing AVOA's optimization efficiency.

In Eq. (3) *w* is a fixed parameter affecting the transition between phases.

Vultures explore less as iterations proceed. When |F| > 1, AVOA enters the exploration phase; if |F| < 1, it switches to exploitation.

3 $$t=\mathrm{h}\times \left({\mathrm{sin}}^{w}\left(\frac{\pi }{2}\times \frac{{iteration}_{i}}{maxiterations}\right)+\mathrm{cos}\left(\frac{\pi }{2}\times \frac{{iteration}_{i}}{maxiterations}\right)-1\right)$$

4 $$F=\left(2\times {rand}_{1}\right)\times z\times \left(1-\frac{{iteration}_{i}}{maxiterations}\right)+t$$

#### Step three: Exploration

In the AVOA framework, vultures use two strategies determined by a parameter, P1, ranging from 0 to 1. A random value, randP1 (0–1), selects the strategy: Eq. (6) if randP1 ≤ P1, else Eq. (8) (as in Eq. 5).

5 $$\mathrm{P}\left(\mathrm{i}+1\right)=\left\{\begin{array}{c}Equation\left(6\right)if{P}_{i} \ge {rand}_{P1}\\ Equation\left(8\right)if{P}_{i} <{rand}_{P1}\end{array}\right.$$

6 $$\mathrm{P}\left(\mathrm{i}+1\right)=\mathrm{R}\left(i\right)-\mathrm{D}\left(i\right)\times F$$

7 $$\mathrm{D}\left(\mathrm{i}\right)=\left|X\times \mathrm{R}\left(\mathrm{i}\right)-\mathrm{P}\left(\mathrm{i}\right)\right|$$

In Eq. (6), vultures randomly forage near a proficient vulture, with distances randomized. P(i) is vulture i's current position, P(i + 1) is its next iteration's position, and F is the satiation rate from Eq. (4). R(i) is a top-performing vulture chosen using Eq. (1), and X is where it moves, determined by X = 2 × rand (uniform random number between 0 and 1).

8 $$P\left(i+1\right)=R\left(i\right)-F+{rand}_{2}\times \left(\left(ub-lb\right)\times {rand}_{3}+lb\right)$$

In Eq. (8), R(i) represents a highly optimal vulture from Eq. (1), F is the saturation rate, and rand2 and rand3 are random variables (0–1). lb and ub generate a random number within their range. rand3 enhances randomness; nearing 1 expands the search environment for diverse spaces.

#### Step four: Exploitation

This strategy is chosen when |F| is less than 1. The exploitation phase has two scenarios: one when |F| is between 0.5 and 1, and another when |F| is less than 0.5. Each scenario involves two distinct approaches.

**Exploitation Scenario 1** “Cautious Feasting” occurs when |F| is between 0.5 and 1. Vultures, hungry but with enough energy for cautious competition, randomly select one of two strategies using a uniform random value, randP2, compared to a predefined parameter, P2 (0 to 1), to adjust the strategy selection likelihood.

9 $$P\left(i+1\right)=\left\{\begin{array}{c}Equation\left(10\right) if {P}_{2}\ge {rand}_{P2} \\ Equation\left(13\right) if {P}_{2}<{rand}_{P2}\end{array}\right.$$

Strategies 1.1: Conflicts.

As mentioned earlier, vultures using this strategy are well-fed (|F| ≥ 0.5) and energetic, clustering around food sources for competitive interactions. Stronger vultures avoid sharing food, while weaker ones tire them out by inciting conflicts, described mathematically by Eqs. (10) and (11).

10 $$P\left(i+1\right)=D\left(i\right)\times \left(F+{rand}_{4}\right)-d\left(t\right)$$

11 $$d\left(t\right)=R\left(i\right)-P\left(i\right)$$

Equation (7) calculates D(i) using vultures' satiation rate, denoted as F in Eq. (4), modified by a random coefficient rand4 (0–1). In Eq. (11), R(i) represents a superior vulture chosen from two groups using Eq. (1) in the current iteration, and P(i) is the vulture's position used to calculate its distance from the best vultures in both groups.

Strategies 1.2: Rotating fly.

Certain vultures exhibit an aerial foraging behavior instead of gathering around food sources on land, characterized by a spiral motion. This behavior is described mathematically using Eqs. (12) and (13). In these equations, R(i) represents the position vector of an optimal vulture in the current iteration, derived from Eq. (1). The functions cos and sin correspond to trigonometric cosine and sine, while rand5 and rand6 are random numbers between 0 and 1. S1 and S2 are calculated using Eq. (12), and Eq. (13) is used to update the vultures' positions.

12 $$\begin{aligned} & S_{1} = R\left( i \right) \times \left( {\frac{{rand_{5} \times P\left( i \right)}}{{2\pi }}} \right) \times {\text{cos}}\left( {P\left( i \right)} \right) \\ & S_{2} = R\left( i \right) \times \left( {\frac{{rand_{6} \times P\left( i \right)}}{{2\pi }}} \right) \times {\text{sin}}\left( {P\left( i \right)} \right) \\ \end{aligned}$$

13 $$P\left(i+1\right)=R\left(i\right)-\left({S}_{1}+{S}_{2}\right)$$

Exploitation Scenario 2: Starvation.

When vultures get hungrier (|F| < 0.5), they become more aggressive, leading to increased competition for food. This involves two strategies randomly selected using Eq. (14), with "randP3" as a random number between 0 and 1, and "P3" as a preset parameter between 0 and 1, influencing the strategy choice.

14 $$P\left(i+1\right)=\left\{\begin{array}{c}Equation\left(16\right) if {P}_{3}\ge {rand}_{P3} \\ Equation\left(17\right) if {P}_{3}<{rand}_{P3}\end{array}\right.$$

Strategies 2.1: Accumulation over the food sources.

Starvation can attract multiple vulture species to a single food source. Equations (15) and (16) model their movement patterns. In Eq. (15), BestVulture1(i) and BestVulture2(i) represent the optimal vultures from two groups in each iteration. The vulture satiation rate, F (calculated in Eq. (4)), and individual vulture positions, P(i), are considered. Equation (16) aggregates all vultures, computing A1 and A2 from Eq. (15), and updating the vulture position to P(i + 1) in the next iteration.

15 $$\begin{aligned} & A_{1} = BestVulture_{1} \left( i \right) - \frac{{BestVulture_{1} \left( i \right) \times P\left( i \right)}}{{BestVulture_{1} \left( i \right) - P\left( i \right)^{2} }} \times F \\ & A_{2} = BestVulture_{2} \left( i \right) - \frac{{BestVulture_{2} \left( i \right) \times P\left( i \right)}}{{BestVulture_{2} \left( i \right)^{2} }} \times F \\ \end{aligned}$$

16 $$P\left(i+1\right)=\frac{{A}_{1}+{A}_{2}}{2}$$

Strategies 2.2: Aggressive competition.

When head vultures become extremely hungry, they lose energy and cannot defend against other vultures. Meanwhile, the remaining vultures become more aggressive in their food search, losing their fear and strategic planning. They approach the head vulture from multiple directions, as described in Eq. (17).

17 $$P\left(i+1\right)=R\left(i\right)-\left|d\left(t\right)\right|\times F\times Levy\left(d\right)$$

Equation (17) uses the variable d(t) to represent the distance between vultures, calculated with Eq. (11). To improve the AVOA algorithm in Eq. (17), we incorporate Levy flight (LF) patterns ^43,44^. The calculation of LFs is based on Eq. (18).

18 $$\begin{aligned} & LF\left( x \right) = 0.01 \times \frac{{{\text{u}} \times \upsigma }}{{\left| v \right|^{{\frac{1}{p}}} }} \\ & \sigma = \frac{{{\text{T}}\left( {1 + \beta } \right) \times {\text{sin}}\left( {\frac{{\pi \beta }}{2}} \right)}}{{{\text{T}}\left( {1 + \beta 2} \right) \times \beta \times 2\left( {\frac{{\beta - 1}}{2}} \right)}} \\ \end{aligned}$$

In Eq. (18), d represents the problem dimensions, u and v are random numbers between 0 and 1, and β is a fixed default value of 1.5.

### Software and simulation framework

The computational environment utilized for this study was Grasshopper, a graphical algorithm editor integrated with Rhino 7^45^. This interactive platform enabled design optimization and iterations.

**Modeling and assembly:** Ventilation bricks, modeled to replicate common real-world designs, were created in Blender, an open-source 3D modeling tool^46^. The room design and façade were then integrated within the Grasshopper environment to enable daylight simulation.

**Figure 3:**
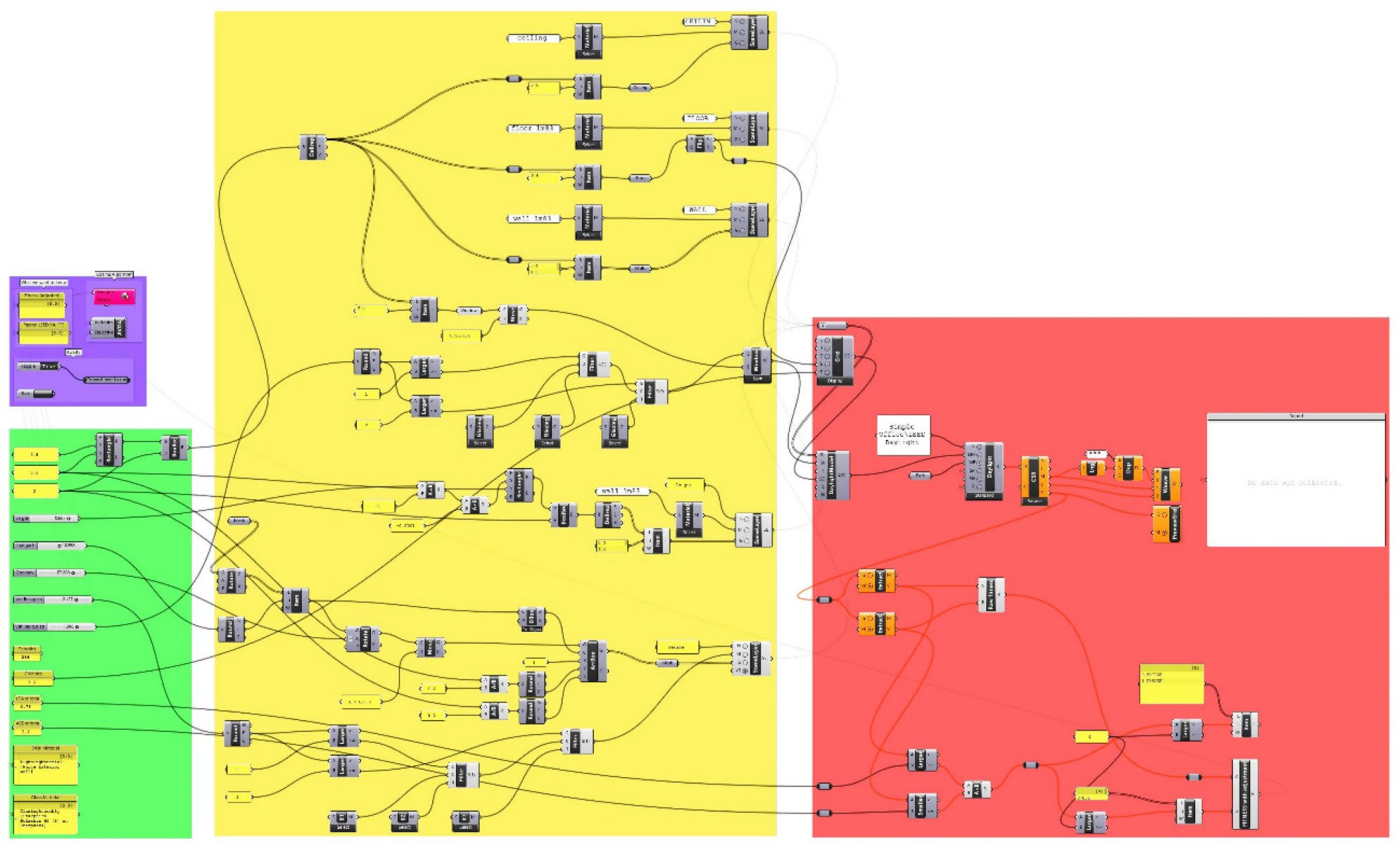
Rhino 7's Grasshopper Blueprint.

**Daylight simulation software:** A validated Radiance ray-tracing system was utilized by the ClimateStudio daylight simulation software to enable high-fidelity computations of daylight and luminance^47^. The scientific validation of the Radiance system lends credibility and precision to the simulation outcomes, as established in prior work^48–50^. As illustrated in Fig. 3, the Grasshopper document encapsulates the complete optimization and simulation workflow.

**Figure 4:**
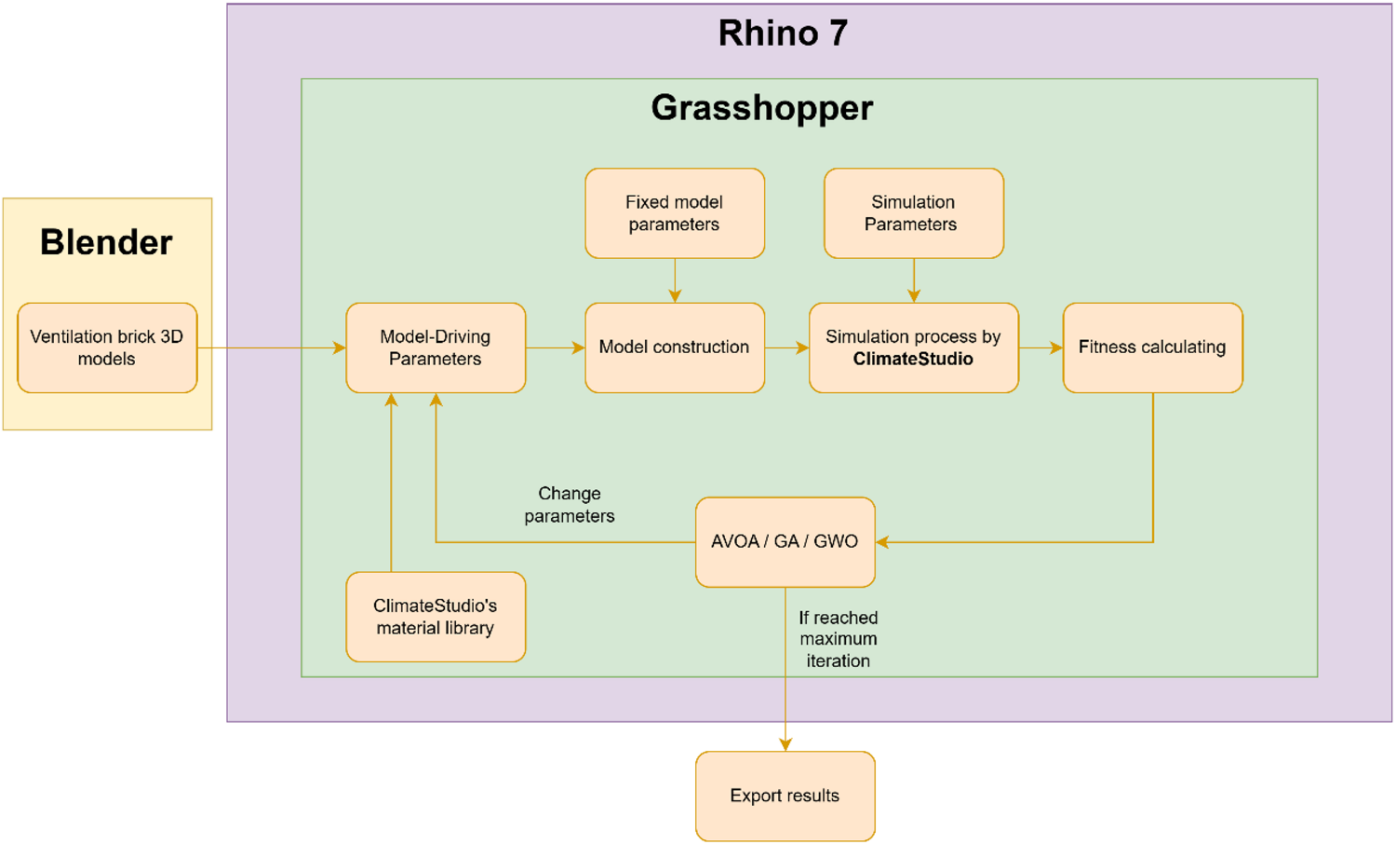
Optimization workflow with software interactions.

Additionally, Fig. 4 presents a simplified flowchart depicting the interactions and roles of the software tools within the workflow.

### Ethical approval

This article does not contain any studies with human participants or animals performed by any of the authors.

## Case study

In order to evaluate the efficacy of the proposed optimization approach, a case study was conducted focusing on a conceptual west-facing room in Long Thanh, Vietnam. The case study aims to demonstrate the capabilities of the methodology in generating facade designs that enhance daylight performance aligned with LEED v4.1 standards.

The case study commences with initial benchmark simulations employing both unshaded and randomly configured facades to establish baseline conditions for comparison. Following this, a series of 45 simulation runs are performed to demonstrate the effectiveness of the proposed optimization technique. In these simulations, various facade parameters such as brick patterns, material types, angular positioning, loggia depth, and glazing assemblies are optimized. This optimization is achieved through the application of the African Vulture Optimization Algorithm (AVOA), strategically aimed at maximizing the differential between Spatial Daylight Autonomy (sDA) and Annual Sunlight Exposure (ASE).

Additionally, this research enhances the understanding of the AVOA's performance in this setting by comparing it with two established metaheuristic algorithms: the Genetic Algorithm (GA) and the Grey Wolf Optimizer (GWO). This comparative aspect is intended to present a brief overview of the relative performance of AVOA in optimizing facade designs, particularly with regard to its effectiveness in achieving a balance between daylight sufficiency and glare control, aligned with the LEED v4.1 standards.

The outcomes of this study are examined from a statistical perspective. This analysis is essential in highlighting the improvements facilitated by the optimization process in contrast to baseline models. Moreover, the analysis includes a review of the optimized design solutions. This review aims to identify consistent material and design choices that notably enhance daylight metrics and compliance with LEED standards, offering valuable guidance for architects and designers in pursuing sustainable and effective facade design strategies.

**Figure 5:**
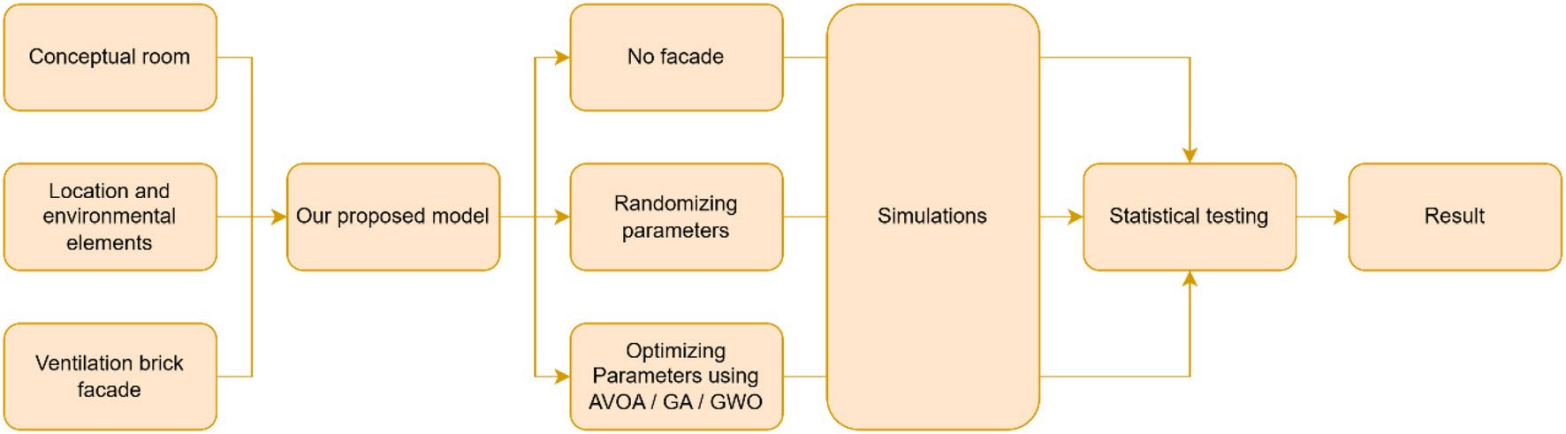
Flowchart of the overall research methodology.

Visualizations comparing the progression of facade configurations across percentile ranges supplement the quantitative analysis. Figure 5 shows the flowchart of the overall research methodology.

By methodically assessing the outcomes against established baselines, this case study provides evidence for the viability of the optimization methodology in enhancing daylighting design practices aligned with green building standards. The knowledge garnered also offers practical insights into facade features conducive to excellent daylight performance.

### Designing the simulation model

#### Room specifications

**Figure 6:**
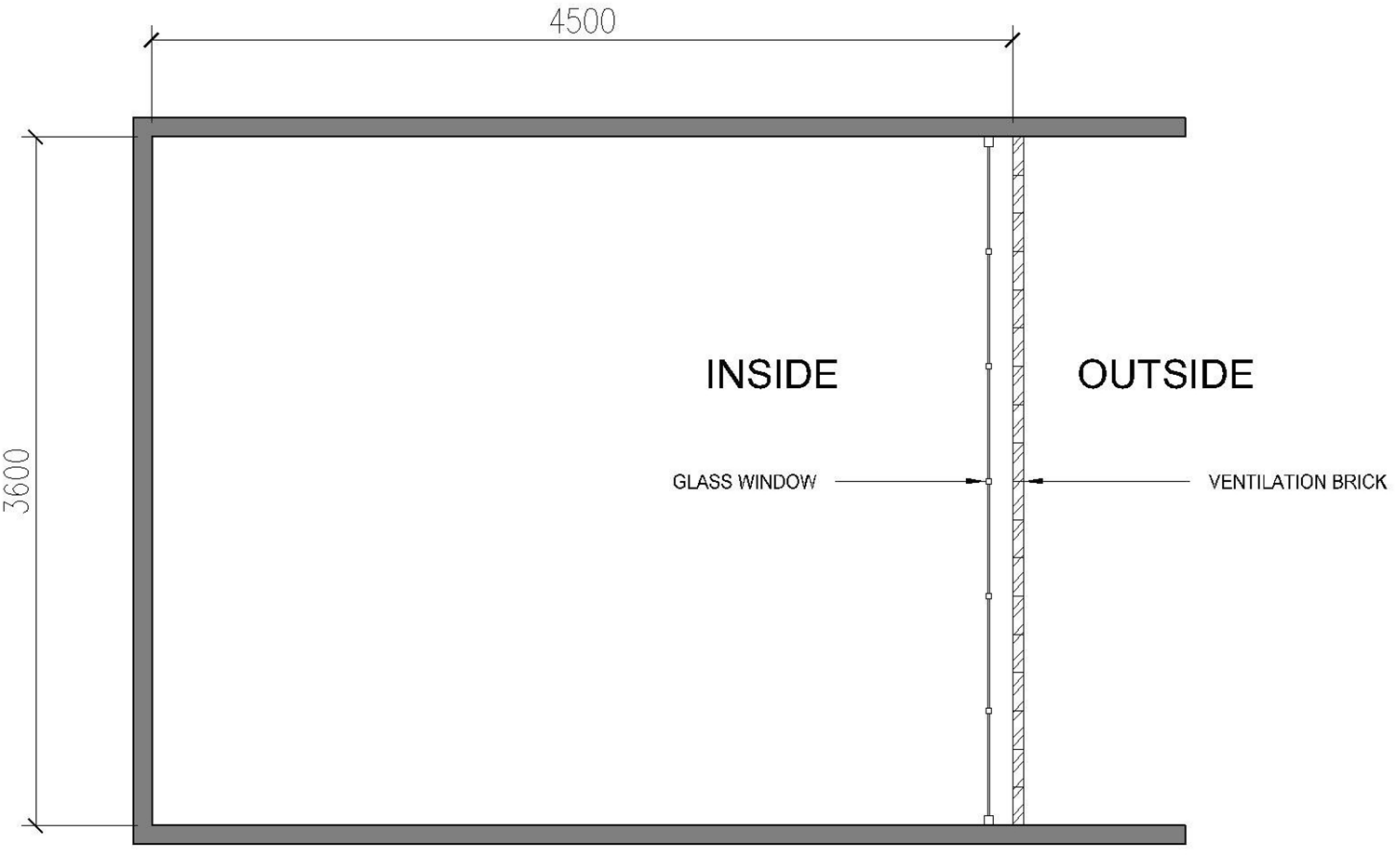
Floor plan of the simulation space.

The simulation space is conceptualized as a room with dimensions measuring 4.5 m in the East–West direction, 3.6 m in the North–South orientation, and a height of 3 m. Predominantly, the western wall features a large glazed window, providing a distinctive daylight interface as shown in Fig. 6. To ensure the consistency and validity of the simulation, no interior furniture was introduced. Material selection for the wall, floor, and ceiling strictly adheres to the LM-83 guidelines established by the IES^5^ (Table 1).

**Table 1:** Model parameters (fixed).

Parameter	Value	Units/type
Space width	3.6	m
Space length	4.5	m
Space height	3	m
Window width	3.6	m
Window height	3	m
Ceiling material	CEILING LM83	–
Wall material	WALL LM83	–
Floor material	FLOOR LM83	–
Loggia material	WALL LM83	–

**Materiality** Materials were selected from ClimateStudio's library^51^, which contains scans of real-world samples to emulate authentic optical properties **(**Table 2**).**

**Table 2:** Materials.

Material name	Type	Reflectance (%)	Specular (%)	Diffuse (%)	R	G	B	Roughness
Wall LM83	Matte	50.00	0.00	50.00	0.5	0.5	0.5	0
Ceiling LM83	Matte	70.00	0.00	70.00	0.7	0.7	0.7	0
Floor LM83	Matte	20.00	0.00	20.00	0.2	0.2	0.2	0
Red Brick 2	Glossy	18.40	0.03	18.37	0.351	0.13	0.054	0.3
Concrete Exterior Wall	Glossy	33.42	0.08	33.33	0.354	0.33	0.279	0.3
White Exterior Wall	Glossy	69.68	0.27	69.41	0.703	0.695	0.651	0.2

**Simulation parameters** A sample count of 256 and grid resolution of 0.1 m (Table 3) were chosen to balance computational efficiency and accuracy within the constraints of our available computing hardware.

**Table 3:** Simulation parameters (fixed).

Parameter	Value	Units/criteria
Sample number per sensor	256	–
Grid size	0.1	m
LEED sDA	≥ 75	%
LEED ASE	≤ 10	%

#### Loggia design

A distinctive architectural feature, the loggia, is also integrated into the room design. The loggia depth, ranging from 0 to 0.8 m, serves as the first parameter in this study and is designed using materials congruent with the walls **(**Table 4**).**

**Table 4:** Model-driving parameters.

Parameter	Range/value	Units/type
Loggia depth	0–800	mm
Brick type	0–23	Integer
Brick angle	− 90 to + 90	Degrees
Brick material	0; 1; 2	0 = Red brick; 1 = Concrete Exterior Wall; 2 = White Exterior Wall
Glass material	0; 1; 2	0 = Starphire; 1 = Solarban 60 (3) on Starphire; 2 = Solarban 72 (3) on Starphire

#### Facade construction

**Figure 7:**
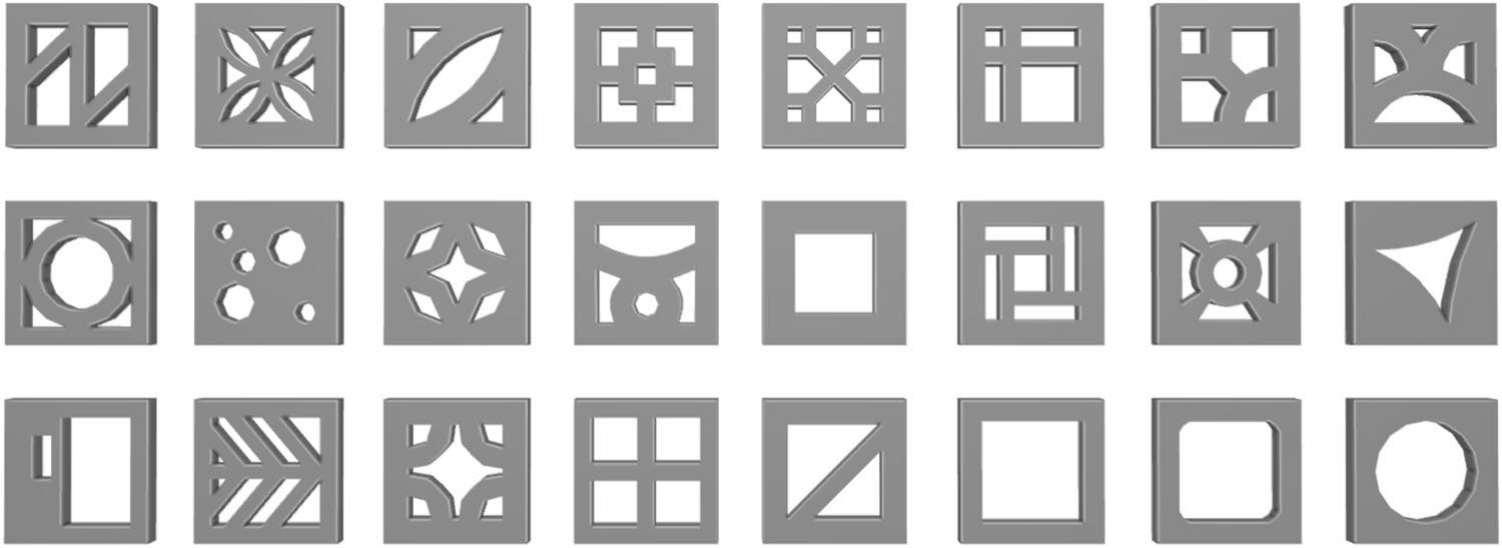
Illustration depicting the various types of ventilation bricks employed in this research study.

Our façade's most prominent feature is the use of ventilation bricks, with dimensions of 200 × 200 × 60 mm. We created 3D models for 24 distinct brick designs that are prevalent in the Vietnamese market, as shown in Fig. 7. The type of brick pattern serves as the second parameter in this study, summarized in Table 4.

The brick material composition constitutes the fourth parameter, with options for red brick, white concrete, and grey concrete. The façade is designed as a wall matching the dimensions of the room's west side. A key engineering capability allows each brick column to be positioned at angles ranging from − 90° to + 90° relative to the original orientation, representing the third design parameter.

#### Glazing

An adjacent glass window is situated inward relative to the façade. Three distinct glass materials have been shortlisted as options for this component, as summarized in Table 5*.* The specific glass composition utilized serves as the fifth and final parameter included in the simulations.

**Table 5:** Glazing assemblies.

Material name	Layer	Tvis (%)	Rvis.front (%)	Rvis.back (%)
Starphire	Single	91.10	8.20	8.30
Solarban 60 (3) on Starphire	Double	74.40	12.10	11.10
Solarban 72 (3) on Starphire	Double	60.60	10.20	11.40

#### Site specificity

The location data from Long Thanh, Vietnam was used due to its specific climatic conditions and daylight variations.

### Benchmark setup for facade optimization

Expanding our understanding of facade optimization techniques can be accomplished by performing reference or benchmark simulations. These simulations offer a basis for evaluating the efficacy of different optimization methods. By comparing the results to a baseline and a simple replication of conventional practices, we can contextualize and quantify the improvements achieved through optimization.

#### Basic unshaded model

The unshaded model omits shading strategies, like facades, retaining only the fundamental window glazing assembly chosen without comprehensive analysis of factors like daylighting and glare reduction. This model establishes a baseline, reflecting performance without intentional design decisions.

#### Randomized model

To replicate conventional facade design practices, we employ a randomized approach. This model utilizes an alternative methodology in which facade parameters are randomly selected, rather than optimized. This design approach prioritizes aesthetics over specific optimization objectives, resulting in notable variability in performance metrics.

#### Repeat simulations

We conducted 45 simulation runs for each model, following established statistical methodology to enhance result reliability. Multiple iterations were employed to mitigate anomalies, thus improving validity. This approach enhances the robustness of our benchmark data, contributing to its utility in informing future facade optimization research.

### Optimization runs

#### Initialization

The initialization procedure for each execution of our metaheuristic optimization employed a set of randomized facade designs. This methodology was selected to promote diversity within the solution space and mitigate potential biases or assumptions that could steer the optimization process.

**Computational limitations** Due to computational constraints imposed by the hardware available, the implementation of the algorithm utilized a modest population size of 10 individuals evolved over just 5 generations. While this abbreviated implementation may seemingly restrict the full capabilities of the algorithm, it represents an authentic, real-world usage scenario. It is important to recognize that daylight simulation is computationally demanding, placing considerable requirements on computing resources. As high-performance computing capabilities are not universally accessible, if the proposed methodology exhibits efficacy despite such stringent limitations, it would suggest robust adaptability to pragmatic conditions encountered in practice.

#### Objective function: maximizing |sDA-ASE|

This research aims to optimize the difference between two key daylight metrics: Spatial Daylight Autonomy (sDA) and Annual Sunlight Exposure (ASE). sDA indicates the percentage of a space receiving at least 300 lx of daylight for over half of occupied hours annually. Higher sDA reduces lighting energy use and improves occupant well-being. ASE identifies overlit areas receiving over 1000 lx of direct sunlight for more than 250 h annually. While sDA and ASE offer valuable insights individually, their combined analysis through the absolute difference |sDA − ASE| provides a more holistic understanding of balanced daylight quality. The idea of using the |sDA − ASE| metric was inspired by the 2018 study by Tabadkani et al.^15^ which demonstrated the value of optimizing the balance between sufficient daylight and minimizing overlighting. Maximizing the |sDA − ASE| difference promotes sufficient daylight while minimizing overlighting discomfort, going beyond simple compliance to enhance the occupant experience through optimized daylighting design.

#### Objective function modifications for LEED V4.1 compliance

Our primary goal is to maximize |sDA-ASE| while also complying with LEED V4.1 criteria. To ensure adherence to LEED standards, solutions that do not meet these criteria have their fitness reduced by multiplying their |sDA-ASE| value by 0.5. This penalty serves two purposes: it indicates the solution's non-compliance while still allowing it to be considered for further refinement or to contribute beneficial traits to the population.

#### Retrieving results

This study takes an unconventional approach by recognizing that the algorithm's optimal solution, based on fitness score, may not always align with aesthetic preferences in architecture. Instead of selecting a single solution, we choose three top-performing solutions from each optimization run to provide designers with a diverse set of options. These selections vary in either brick pattern or material composition, ensuring distinct choices. This approach comprehensively assesses algorithm performance while accommodating subjective design preferences.

### Results

Baseline model: A total of 45 simulations were conducted for the baseline model, revealing consistent results. The Spatial Daylight Autonomy (sDA) was observed to be 1, indicating optimal daylight conditions. However, the Annual Sunlight Exposure (ASE) was measured at 0.9158, signifying excessive sunlight exposure and raising concerns for occupant comfort **(**Table 6**).**

**Table 6:** Case processing summary.

Model	Cases					
	Valid		Missing		Total	
	N	Percent	N	Percent	N	Percent
|sDA − ASE|						
Baseline	45	100.0	0	0.0	45	100.0
Randomized	45	100.0	0	0.0	45	100.0
Optimized	45	100.0	0	0.0	45	100.0

Randomized model: The randomized model, which employed stochastic selection of design parameters, was run for 45 simulations. The mean score for the objective function |sDA − ASE| was approximately 0.494 with a 95% confidence interval ranging from 0.416 to 0.572. A high degree of variability was evident with a standard deviation of 0.261. The observed range for this model spanned from a minimum of 0 to a maximum of 0.8842 **(**Table 7**).**

**Table 7:** Descriptive statistics of |sDA − ASE|.

Model				Statistic	Std. error
|sDA − ASE|	Randomized	Mean		0.493887	0.0388463
		95% confidence interval for mean	Lower bound	0.415597	
			Upper bound	0.572176	
		5% trimmed mean		0.500139	
		Median		0.586000	
		Variance		0.068	
		Std. deviation		0.2605887	
		Minimum		0.0000	
		Maximum		0.8842	
		Range		0.8842	
		Interquartile range		0.3913	
		Skewness		− 0.777	0.354
		Kurtosis		− 0.567	0.695
	OPTIMIZED	Mean		0.936538	0.0049558
		95% confidence interval for mean	Lower bound	0.926550	
			Upper bound	0.946526	
		5% trimmed mean		0.937367	
		Median		0.943900	
		Variance		0.001	
		Std. deviation		0.0332447	
		Minimum		0.8702	
		Maximum		0.9860	
		Range		0.1158	
		Interquartile range		0.0579	
		Skewness		− 0.349	0.354
		Kurtosis		− 0.923	0.695

AVOA-Driven facade optimization model: 45 solutions from the the model utilizing the AVOA displayed a mean objective function value of approximately 0.937 with a tight 95% confidence interval between 0.927 and 0.947. The standard deviation was remarkably low at 0.033, suggesting a strong consistency in the results. The minimum and maximum values for this model were 0.8702 and 0.9860, respectively, with a range of 0.1158 (Table 7).

GA-Driven facade optimization model: The Genetic Algorithm (GA) model yielded an average objective function value of approximately 0.954947, with a slightly higher median of 0.964900, indicating a trend towards better-performing solutions. The results were consistent, with a low variance of 0.001 and a standard deviation of 0.0293950, indicating a concentration of data points around the mean. The range of these values was between 0.8772 and 1.0000 (Table 8).

**Table 8:** Descriptive statistics of |sDA − ASE| by GA and GWO.

Model				Statistic	Std. error
|sDA − ASE|	GA	Mean		0.954947	0.0043819
		95% confidence interval for mean	Lower bound	0.946115	
			Upper bound	0.963778	
		5% trimmed mean		0.956806	
		Median		0.964900	
		Variance		0.001	
		Std. deviation		0.0293950	
		Minimum		0.8772	
		Maximum		1.0000	
		Range		0.1228	
		Interquartile range		0.0420	
		Skewness		− 0.954	0.354
		Kurtosis		0.652	0.695
	GWO	Mean		0.963673	0.0046375
		95% confidence interval for mean	Lower bound	0.954327	
			Upper bound	0.973020	
		5% trimmed mean		0.966178	
		Median		0.975400	
		Variance		0.001	
		Std. deviation		0.0311094	
		Minimum		0.8772	
		Maximum		1.0000	
		Range		0.1228	
		Interquartile range		0.0491	
		Skewness		− 0.987	0.354
		Kurtosis		0.609	0.695

GWO-Driven facade optimization model: The Grey Wolf Optimizer (GWO) model demonstrated a mean objective function value of about 0.963673, with a higher median of 0.975400, suggesting a bias towards more effective solutions. This model also showed consistent results with a low variance of 0.001 and a standard deviation of 0.0311094. The range of solutions varied from 0.8772 to a maximum of 1.0000, indicating the GWO model's ability to generate highly effective facade optimization solutions (Table 8).

Three statistical tests were conducted to examine differences in the |sDA − ASE| index between models. The sample consisted of 45 randomized models and 45 optimized models.

An independent samples t-test compared the unshaded and randomized models. The randomized models (M = 0.5, SD = 0.3) differed significantly from the unshaded models (M = 0.08), t(88) = 10.5, p < 0.001, d = 1.6.

A second independent samples t-test was conducted between the optimized and unshaded models. Optimized models (M = 0.9, SD = 0.03) showed a significant difference from unshaded models (M = 0.08), t(88) = 172, p < 0.001, d = 25.6.

Finally, an independent samples t-test with equal variances not assumed compared the randomized and optimized models. Levene's test indicated unequal variances between groups, F(1, 88) = 70.452, p < 0.001. The t-test revealed a significant difference between models, t(88.64) = 11.303, p < 0.001, d = 2.38.

**Figure 8:**
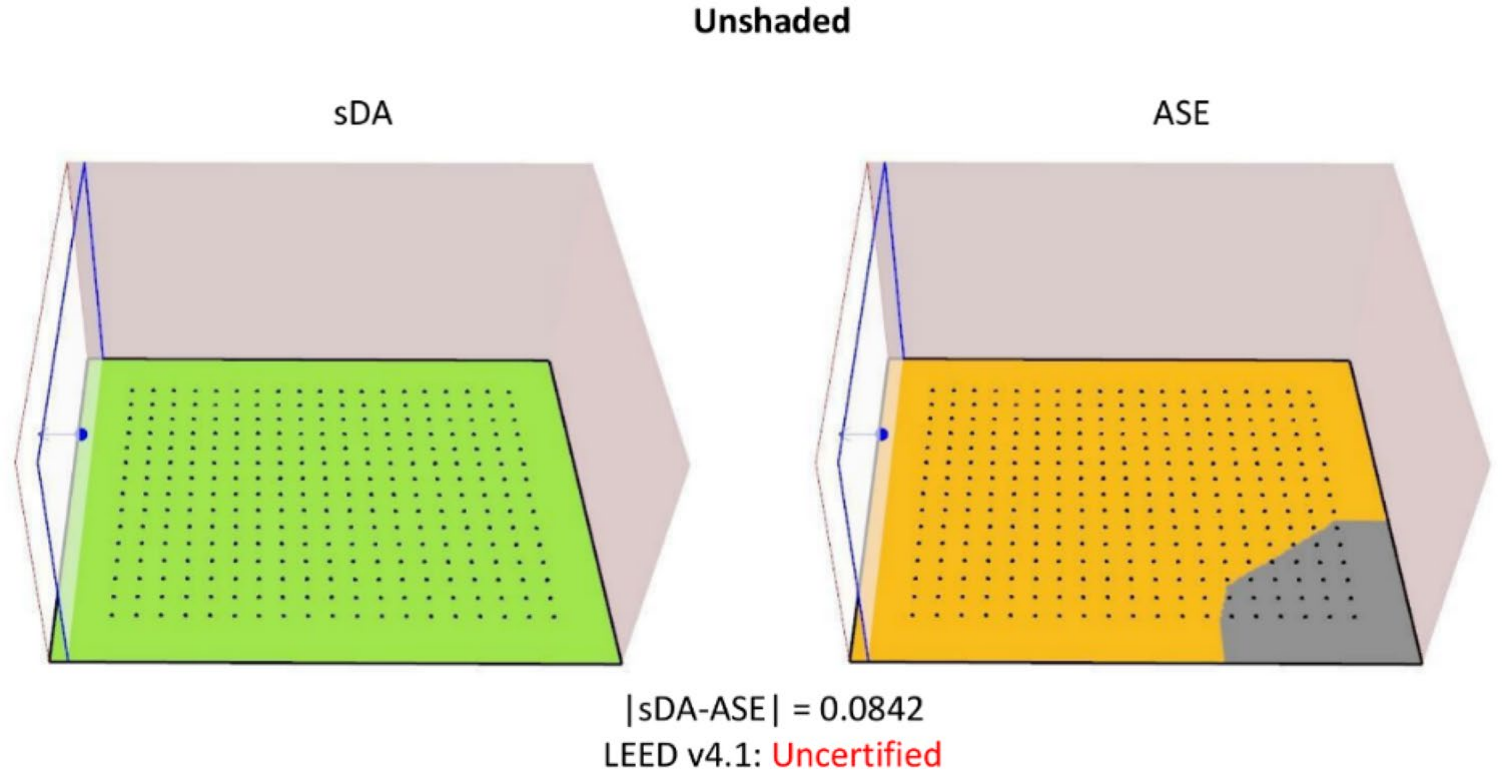
sDA and ASE metrics calculated using an unshaded model, representing idealized baseline conditions.

Unshaded configurations exhibited suboptimal performance, failing to satisfy Leadership in Energy and Environmental Design (LEED) standards. While the Spatial Daylight Autonomy (sDA) achieved an optimal value of 1, the high Annual Sunlight Exposure (ASE) of 0.9158 created unfavorable indoor visual comfort conditions, potentially imposing health risks (Fig. 8)*.*

**Figure 9:**
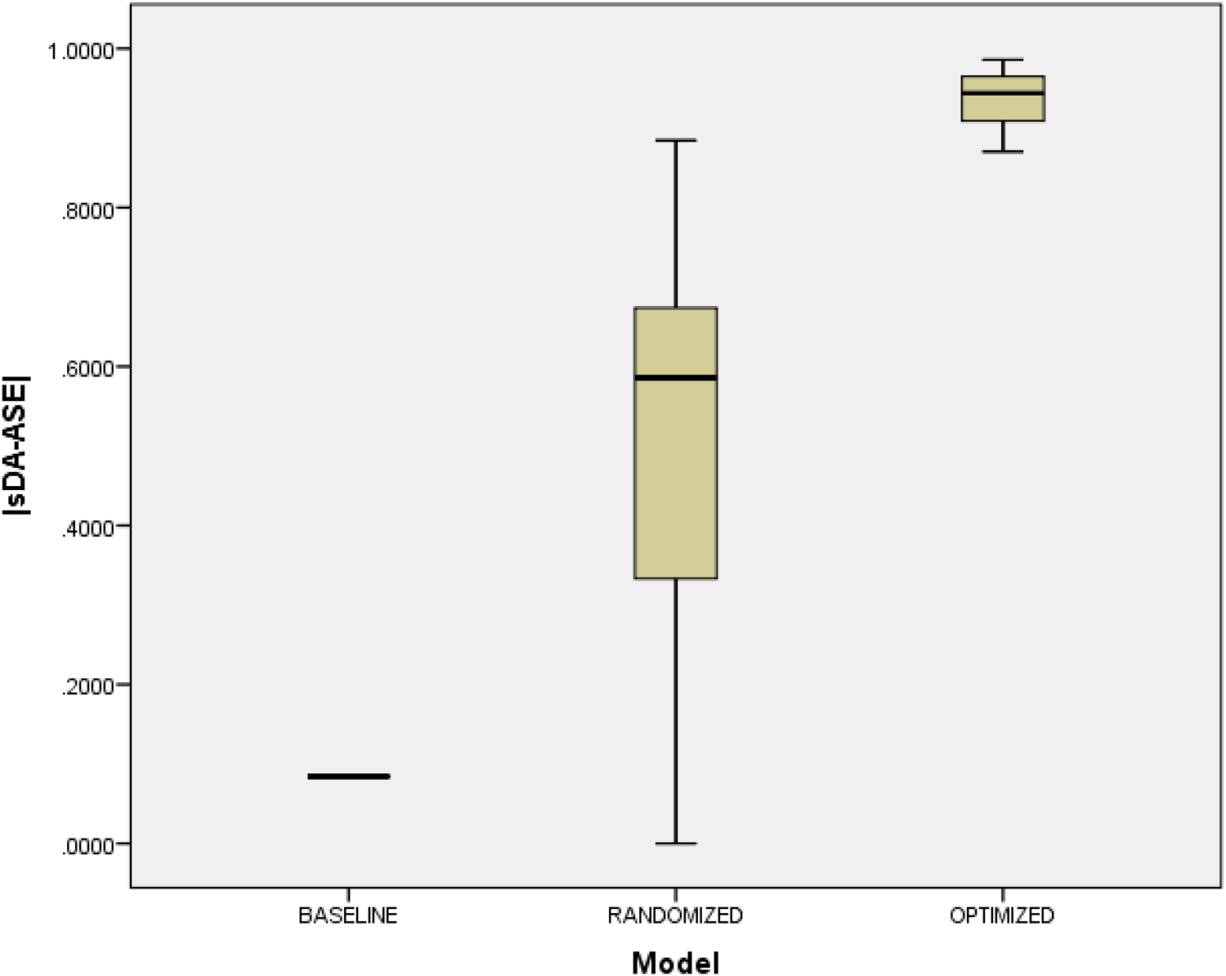
Boxplot displaying distributions of |sDA − ASE| index scores for unshaded models, randomized models, and optimized models.

**Figure 10:**
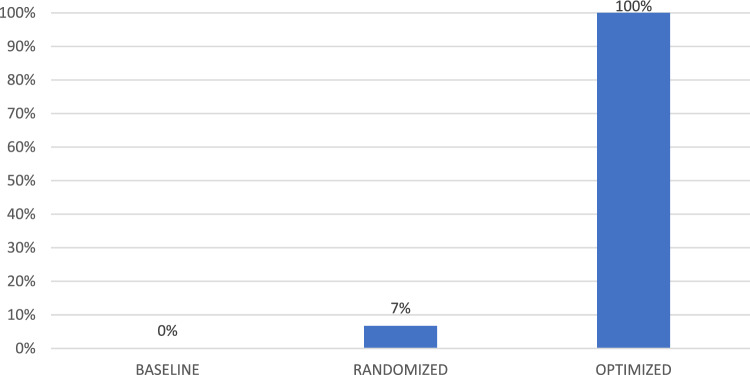
LEED standards compliance by model.

Randomized configurations offered moderate enhancements, as evidenced by a mean absolute difference of 0.4939 between sDA and ASE, contrasted with 0.0842 in unshaded arrangements (p < 0.001). Figure 9 illustrates box plots depicting the differences in sDA and ASE between the randomized and unshaded configurations. However, the increased standard deviation and range signify inconsistency, rendering this approach unreliable. Concerningly, only 6.7% (3 out of 45) of randomized configurations satisfied LEED compliance criteria (Fig. 10). Therefore, arbitrarily selecting this design would likely necessitate substantial post-construction modifications to achieve LEED certification.

Optimized configurations demonstrated superior performance in both fitness metrics (|sDA − ASE|) (p < 0.001) and LEED compliance, achieving a 100% success rate. This not only alleviates certification concerns for architects but also enhances indoor living conditions beyond the scope of LEED standards. Further examination reveals that the algorithm is highly adaptable, incorporating 71% (17 out of 24) of available brick types, as well as all brick materials and glazing assemblies. This versatility allows for a wide selection of design options, effectively solving the problem with multiple viable solutions.

**Figure 11:**
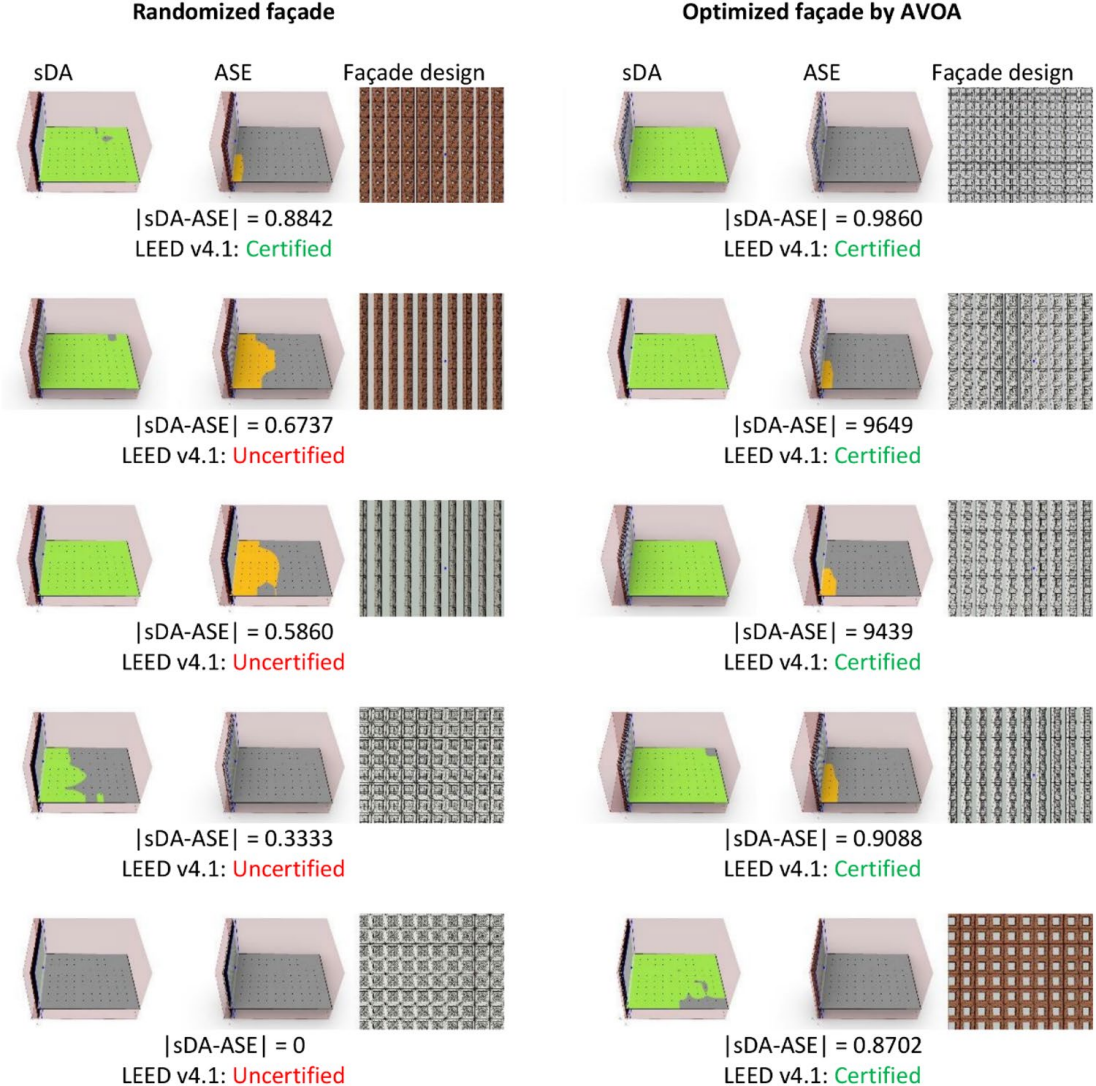
Side-by-side comparisons of the sDA and ASE values for a randomized model and a model optimized using the AVOA algorithm.

Figure 11 visualizes the sDA and ASE metrics along with facade design solutions at various percentiles for both randomized and optimized models. The figure comprises side-by-side rendered images of the facade for each model at the 0th, 25th, 50th, 75th, and 100th percentiles, enabling visual comparison of how the facade solutions evolve across the percentile range.

**Figure 12:**
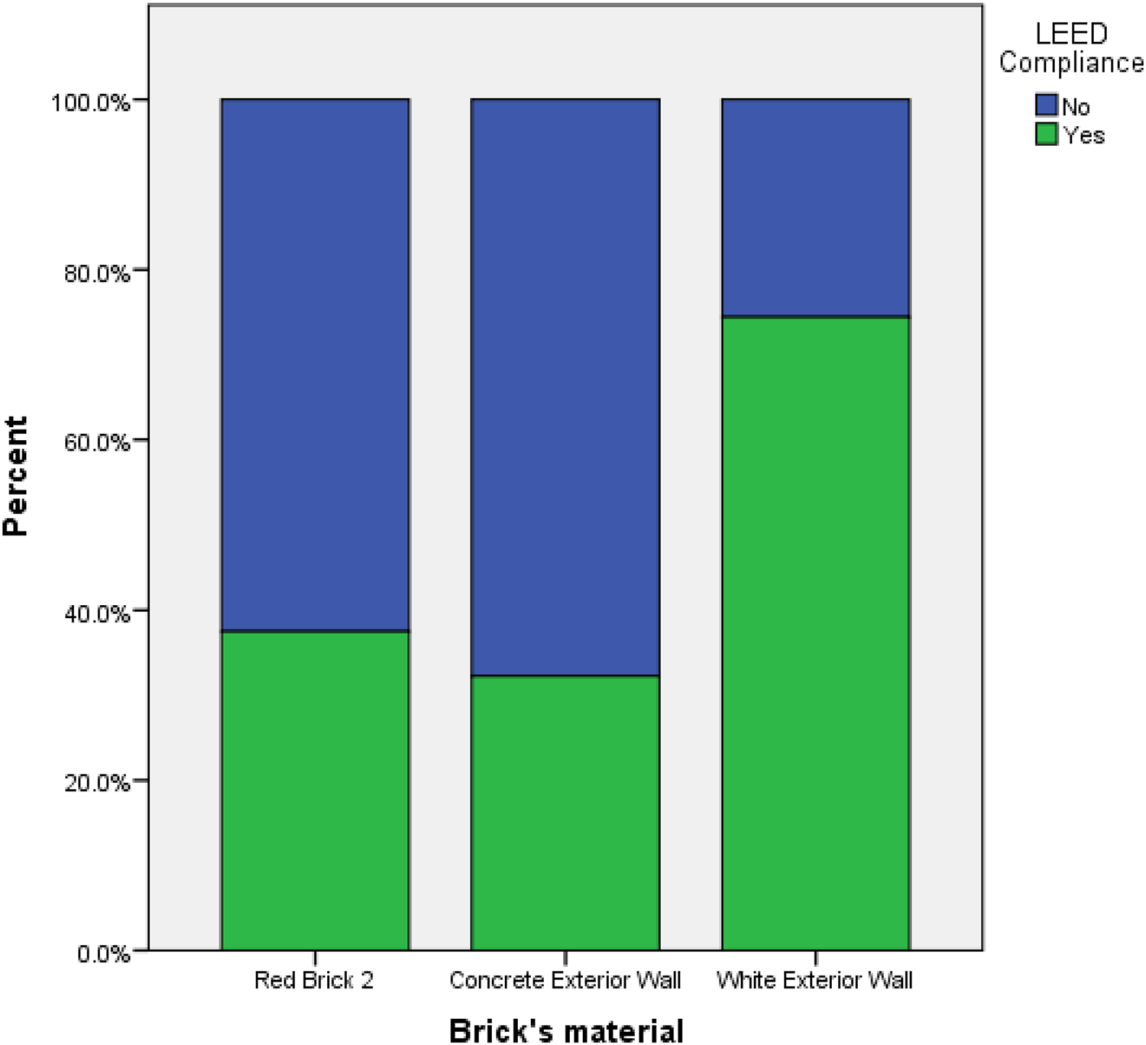
Percentage of LEED compliance grouped by brick material.

**Figure 13:**
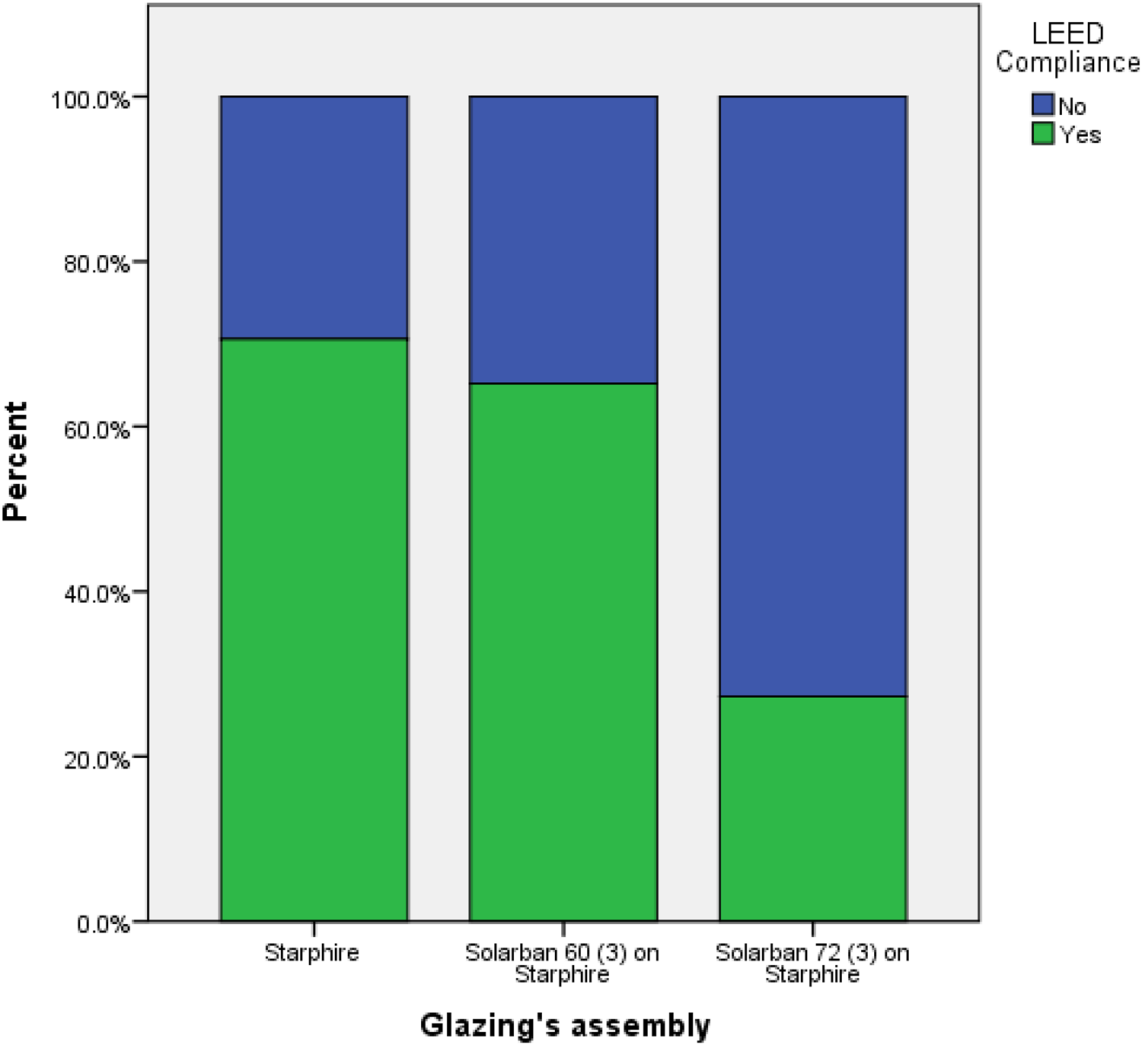
Percentage of LEED compliance grouped by glazing’s assembly.

In a comparative analysis between LEED-compliant and non-compliant groups, statistical evaluations reveal no significant differences in the types of bricks selected (p = 0.072) or in the depth of the loggias (p = 0.349). However, the inclination angle exhibits a higher mean value of 31.11° within the LEED-compliant group, compared to a mean of 4.39° in the non-compliant group (p = 0.003). This discrepancy may be attributed to factors such as glazing orientation and geographical location. Additionally, variations in both brick material (p = 0.001) and glazing assembly (p = 0.001) were observed. Specifically, constructions employing 'White concrete wall' as the brick material had the highest rate of LEED compliance at 74%, compared to those utilizing other materials **(**Fig. 12**).** Regarding glazing assembly, 'Solarban 72 (3) on Starphire' demonstrated the lowest rate of LEED compliance at 27%, whereas 'Solarban' featured the highest at 71% (Fig. 13). These results suggest that lighter-colored bricks and more transparent glazing materials are conducive to achieving superior daylight performance. This may be because lighter bricks and transparent glazings allow more diffuse daylight to permeate into interior spaces, which contributes to higher sDA levels while remaining weak enough to not induce glare and discomfort. The increased indirect lighting enabled by these materials enhances sDA without causing visual or thermal discomfort for occupants.

A comparison of the African Vulture Optimization Algorithm (AVOA) with the Genetic Algorithm (GA) and Grey Wolf Optimizer (GWO) using a one-way ANOVA test revealed significant differences in the mean |sDA − ASE| values (p < 0.001, Table 9). Subsequent post hoc tests indicated that AVOA exhibited a statistically significantly lower mean |sDA − ASE| value (0.936538) compared to both GA (0.954947, p = 0.006) and GWO (0.963673, p < 0.001). However, no significant difference was observed between GA and GWO (p = 0.188). Despite statistical variations in performance, all three algorithms demonstrated good results, with GA and GWO both reaching the maximum possible value of 1. This indicates the high effectiveness of GA, GWO, and AVOA in optimizing facade designs for daylighting, though AVOA comparatively underperformed.

**Table 9:** ANOVA and LSD multiple comparisons of daylight optimization algorithms.

ANOVA						
|sDA−ASE|						
	Sum of squares	df	Mean square	F	Sig	
Between groups	0.017	2	0.009	8.820	0.000	
Within groups	0.129	132	0.001			
Total	0.147	134				

Multiple comparisons						
Dependent variable	|sDA − ASE|					
LSD						
(I) Model		Mean difference (I–J)	Std. error	Sig	95% confidence interval	
					Lower bound	Upper bound
AVOA	GA	− 0.0184089*	0.0065964	0.006	− 0.031457	− 0.005361
	GWO	− 0.0271356*	0.0065964	0.000	− 0.040184	− 0.014087
GA	AVOA	0.0184089*	0.0065964	0.006	0.005361	0.031457
	GWO	− 0.0087267	0.0065964	0.188	− 0.021775	0.004322
GWO	AVOA	0.0271356*	0.0065964	0.000	0.014087	0.040184
	GA	0.0087267	0.0065964	0.188	− 0.004322	0.021775

*The mean difference is significant at the 0.05 level.

**Figure 14:**
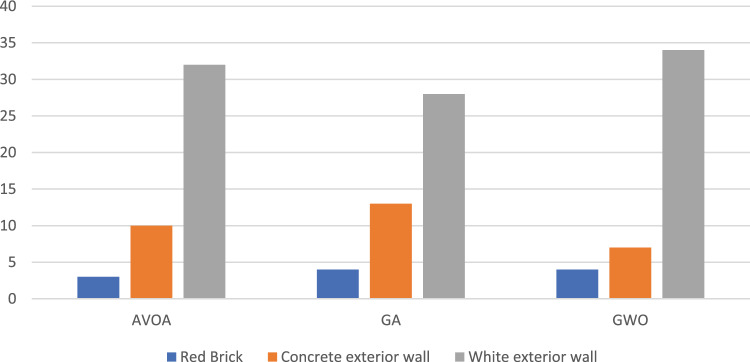
Comparison of brick material selection across optimization algorithms.

**Figure 15:**
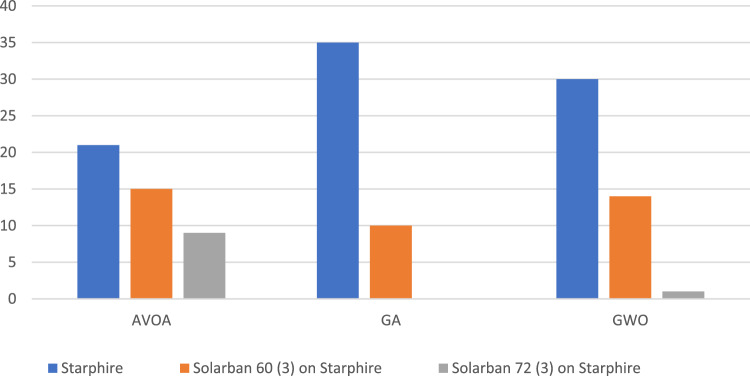
Comparison of glass material selection across optimization algorithms.

**Figure 16:**
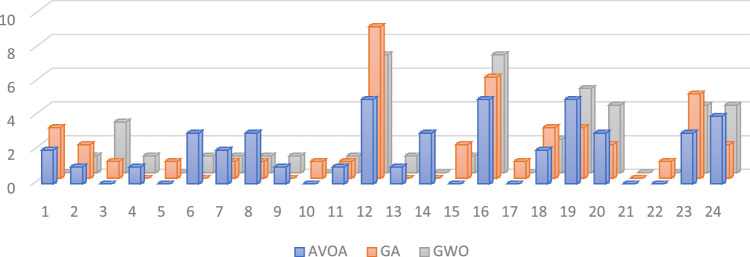
Comparison of brick type selection across optimization algorithms.

Investigating the design choices of the algorithms reveals no significant differences in the depth of the loggias used in their solutions (p = 0.203). The GWO utilized significantly more neutral angles compared to the AVOA (10.16° versus 31.80°, p = 0.01), suggesting a more balanced solution pool from GWO in terms of bricks' orientation. All three algorithms successfully employed all the brick materials, with a preference for lighter-colored bricks, demonstrating consistency (Fig. 14). Regarding the glass materials, all three models showed a preference for more transparent glass. However, AVOA provided more solutions with darker glass, expanding its suitability for certain aesthetic preferences (Fig. 15). In terms of brick pattern types, all three algorithms utilized over 70% of all brick types. Combined, only one brick type was not chosen by any algorithm, once again demonstrating the diversity in the solution space that the algorithms can achieve (Fig. 16).

In conclusion, all three algorithms achieved very good daylight performance and diverse designs. Surprisingly, the AVOA exhibited lower performance compared to the other two algorithms. However, it offered more design choices with darker glass, which could be advantageous for certain aesthetic preferences. The reduced performance of the AVOA might stem from its limited iterations and population size, or the specific nature of the solution space in this scenario. Conducting additional tests and incorporating more algorithms would be beneficial to gain further insights into the underlying factors influencing these outcomes.

### Limitations

While our study demonstrates the efficacy of our approach when using basic computational resources such as a consumer laptop, it is important to acknowledge potential constraints. We did not assess how the method scales with larger datasets or more complex scenarios that might necessitate advanced computational power.

Our study introduced a model that demonstrates flexibility and adaptability with a degree of modularity and parametric flexibility. Because of this design approach, it's adaptable to various building types and environmental contexts. Moreover, it can integrate added conditions like trees, ground elements for lower-level rooms, or nearby structures. However, it is imperative to recognize the model's limitations. Despite its adaptability, it may not be optimally suited for designing façades for buildings with intricate shapes or for scenarios that encompass multiple rooms or an entire level. Such situations could necessitate modifications to the model. Additionally, to ensure its broad applicability and to fit within the hardware constraints of our research, we intentionally kept the model simple.

In terms of specific application context, the research was particularly oriented towards daylighting simulations in a location in Vietnam. The model adopted a westward orientation and made use of a relevant regional weather dataset. Our choice in brick types, their materials, and glazing materials were primarily influenced by what's readily available in the market and our practical experience. Although this decision aimed to mirror standard construction practices, it constrains the study to materials that are readily available and commonly used instead of exploring a broader spectrum of options.

It's also essential to recognize that while we employ scientifically validated simulations, these tools may not fully mirror the complexities of the real world. Empirical testing in tangible settings can offer insights that simulations might miss.

Moreover, the cornerstone of our research consists of the Spatial Daylight Autonomy (sDA) and Annual Sunlight Exposure (ASE) metrics from the Leadership in Energy and Environmental Design (LEED) version 4.1 standards. As metrics and standards progress over time, maintaining adaptability and openness to newly developed or refined metrics in future studies is imperative.

## Conclusion and future research

This study proposes an approach for optimizing west-facing room façades to enhance daylight performance and align with LEED v4.1 sustainability standards. The methodology integrates parametric modeling, metaheuristic optimization, and validated daylight simulations.

The results demonstrate the efficacy of the African Vulture Optimization Algorithm in generating design solutions that achieve 100% LEED compliance while maximizing the difference between sDA and ASE. In contrast, randomized models only attained 6.7% LEED compliance. Statistically, optimized configurations outperformed unshaded and randomized models on the fitness metric.

Analysis of materials contributing to LEED compliance revealed lighter-colored bricks like white concrete and more transparent glazing to be advantageous. The algorithm exhibited versatility by successfully utilizing 71% of available brick types and all material options. This adaptability enables architectural flexibility to accommodate aesthetic preferences.

While promising, certain limitations exist, including constraints imposed by computational resources. Additionally, further validation through empirical testing can enrich these simulation-based findings.

In terms of algorithmic performance, our comparative analysis revealed distinct differences. The AVOA, while offering a broad range of design choices, showed a lower mean |sDA-ASE| value compared to GA and GWO, suggesting a slightly reduced effectiveness in optimizing for daylight performance. However, all algorithms, including GA and GWO, demonstrated high effectiveness in achieving optimal facade designs, with GA and GWO performing comparably. These insights into algorithmic performance are crucial for future applications in facade design optimization.

In summary, this research proposes an optimization technique for daylighting design that integrates metaheuristic algorithms, parametric modeling, and simulation while aligning with LEED standards. The method demonstrates efficacy in generating design solutions that balance daylight sufficiency, minimize discomfort glare, and fulfill sustainability requirements. This offers architects enhanced flexibility and performance optimization capabilities, contributing to the advancement of daylight-integrated façade design practices.

As we look towards future research, there are several areas for exploration and validation.

- Further iterations and comparisons with other techniques are recommended to provide a richer comparative analysis. Specifically, contrasting metaheuristic with traditional optimization methods might yield important insights.
- Selecting metaheuristic algorithms with strong empirical evidence is advised.
- An encompassing approach that evaluates both environmental and human-centric aspects can expand our view of building optimization.
- Adapting the model to diverse building types and integrating it with real-time systems can be advantageous.
- Considering diverse geographical locations, weather datasets, building orientations, and construction materials and techniques is essential for a comprehensive understanding.

## Data Availability

Upon request and subject to reasonable conditions, the corresponding author can provide the data, model, or code that underlie the findings of the study.
